# Online, Face-to-Face, or Blended Learning? Faculty and Medical Students' Perceptions During the COVID-19 Pandemic: A Mixed-Method Study

**DOI:** 10.3389/fmed.2022.791352

**Published:** 2022-02-03

**Authors:** Hani Atwa, Mohamed Hany Shehata, Ahmed Al-Ansari, Archana Kumar, Ahmed Jaradat, Jamil Ahmed, Abdelhalim Deifalla

**Affiliations:** ^1^Medical Education Unit, College of Medicine and Medical Sciences, Arabian Gulf University, Manama, Bahrain; ^2^Medical Education Department, Faculty of Medicine, Suez Canal University, Ismailia, Egypt; ^3^Department of Family and Community Medicine, College of Medicine and Medical Sciences, Arabian Gulf University, Manama, Bahrain; ^4^Department of Family Medicine, Faculty of Medicine, Helwan University, Helwan, Egypt; ^5^Department of Physiology, Sri Ramachandra Medical College, SRIHER, Chennai, India; ^6^Department of Anatomy, College of Medicine and Medical Sciences, Arabian Gulf University, Manama, Bahrain; ^7^Department of Human Anatomy and Embryology, Faculty of Medicine, Suez Canal University, Ismailia, Egypt

**Keywords:** face-to-face, online, COVID-19 adaptations, blended learning, COVID-19 experience

## Abstract

**Background:**

COVID-19 pandemic forced educational institutions to adopt online methods which were inevitable to keep continuity of education across all academia after suspension of traditional educational systems. The aim of this study was to explore the experience of faculty and students of online and face-to-face learning, and their preference of the mode of learning after the pandemic.

**Methods:**

This is a mixed-method study. Quantitative data was collected through a survey from 194 medical students and 33 faculty members, while qualitative data was collected through two focus group discussions with 9 students and another two with 13 faculty members. Quantitative variables were presented as means and standard deviations. Paired samples *t*-test and Chi-square test were used. Thematic analysis of qualitative data was used to code, interpret, and make sense of data.

**Results:**

Mean scores of responses of faculty members and students were higher for face-to-face and blended learning compared to online learning in all survey statements with statistically significant differences. More than half of the students (53.1%) preferred the face-to-face mode of learning, while most of the faculty members (60.6%) preferred the blended mode of learning. Qualitative analysis identified five themes, namely: “Transforming the way theoretical teaching sessions are given,” “Face-to-face teaching at campus cannot be replaced for some types of education,” “Interaction in online sessions is limited,” “Problems and challenges of online examinations,” and “Technical issues and challenges of online education.” It revealed suggestions that at least 30% of the curriculum could be taught online post-COVID-19. Some aspects of clinically oriented teaching including history taking and case discussions can also be delivered online in the future. Faculty members and students reported that dealing with online education was not difficult, although the transition was not smooth.

**Conclusion:**

Medical students and faculty members were in favor of face-to-face and blended modes of learning. However, they perceived online mode of learning as an acceptable adaptation in theoretical teaching and in some clinically oriented teaching including history taking and clinical case discussions. Although face-to-face education in medicine is irreplaceable, the blended mode of learning remains an acceptable and practical solution for the post-COVID era.

## Introduction

The COVID-19 pandemic affected the normal functioning of all academic institutions globally (1). Health professions educational institutions were disturbed to the maximum, obviously more than other education institutions. This is mainly because of the inherent nature of teaching and learning in such institutions, which depends mainly on the contact between the teachers, students, and patients in training sites (2, 3).

Lockdown measures were implemented in all countries, which forced educational institutions to search for alternatives for continuing their educational programs without compromising the safety of their students and teachers (4–6). Very soon, it became apparent that the judicious use of technology could solve many of the problems, and therefore, almost all educational institutions initiated a paradigm shift in their policies to rapidly introduce online methods for teaching as well as assessment (7–10). Adapting online methods was inevitable to keep the continuity of education across all academia (11–13). Because of the nature of the rapid transition, most of the medical education institutions started with adopting an asynchronous approach by preparing PowerPoint presentations with voiceovers and sharing them with the students via emails or social media applications. Medical schools also used Learning Management Systems (LMS) to upload reading material, videos of physical examination, quizzes, and presentations to engage students in these asynchronous learning activities. LMS as well as other platforms enabled institutions to use other asynchronous engaging modalities like discussion forums, in which students could initiate discussions or post questions on the forums and later a faculty can respond or guide at their convenience (1, 4, 6, 14). As medical education institutions developed expertise, ensured the necessary infrastructure, and guaranteed access to online meeting platforms, they started to adopt more synchronous methods where students and teachers are engaged at the same time giving space for more explanations and active discussions. A mixed model of using both modalities (synchronous and asynchronous) was also adopted from the start by some medical education institutions or maintained in later stages to maximize the benefits of each modality (14, 15).

Medical schools witnessed additional responsibility to continue with the curriculum, to enable timely graduation of the students of 2020, in order to support the overwhelmed healthcare systems battling the pandemic (16, 17). The College of Medicine and Medical Sciences at the Arabian Gulf University (CMMS-AGU) was among the first institutions in the region that embraced this transition systematically (18). Before the pandemic, teaching was conducted completely face-to-face on campus. Immediately after the pandemic started, the CMMS-AGU started employing the asynchronous approach, where the teachers started to record the lectures and demonstrations and upload them on a LMS (Moodle) for their students who have access to view and download them. Then, arrangements were done to allow the teachers to have synchronous sessions with the students, through online meeting platforms, where student-to-teacher and student-to-student interaction was live, and teachers could respond in a timely fashion to students' questions and requests. However, the good practice of sharing reading material and quizzes via the LMS was maintained parallelly with the synchronic sessions. Toward the end of the academic year 2019–2020 (under the pandemic-related restrictions), the College decided to conduct written exams via an online application for all students in all phases of the medical program, namely premedical phase (year 1), pre-clerkship phase (years 2–4), and clerkship phase (years 5 and 6). For performance exams, different decisions were taken according to the phase of students and the objectives of each examination. Some examinations were postponed for the following academic year, while some were conducted online (19).

Soon after it was possible for the teachers to go to the campus (while students' attendance was still suspended), they could demonstrate practical (lab) sessions and some clinical skills (simulation) sessions from campus and livestream them to the students who were off campus. Such sessions were also video recorded and uploaded on Moodle for viewing later by the students. Starting from September 2021, students and teachers attend at campus and clinical training settings and teaching is done in a blended manner, where the theoretical teaching is done online while practical and clinical teaching is done face-to-face. At that stage the decision was to have all student assessment activities on campus.

To guarantee the readiness of CMMS-AGU faculty for online teaching and assessment, all full-time and adjunct teachers were trained through a series of hands-on workshops (with strict adherence to COVID-19 prevention guidelines) on using technology in teaching and modalities of online assessment. In addition, several instructional videos on technology-enhanced teaching were created and shared with them (16, 20).

Few studies have already documented the preferences of students for online or face-to-face learning for different reasons. Paechter and Maier (21) and Paechter et al. (22) found that the students preferred online learning for providing well-structured learning materials and enabling studying from home at their own pace and convenience. At the same time, they also liked face-to-face learning for specific reasons like acquiring motor skills and establishing interpersonal relations. Moreover, Muthuprasad et al. (23) reported that in courses that are more practical/skill oriented, changing entirely to online mode may not be a viable option and such institutions ought to design a hybrid/blended curriculum involving both face-to-face and online methods.

Regarding the quality of achievement of the learning outcomes in online and face-to-face learning in general and non-medical education courses, there are diverse opinions. Some authors found that achievement of the student learning outcomes is less efficient in online learning than in face-to-face learning (24–26), while others reported no significant difference when compared to face-to-face teaching (27–29). For medical courses, however, online learning might be more suitable for students in the pre-clerkship phases compared to students in the clerkship phase. While clinical reasoning and approach to clinical problems can be taught via online media, teaching of physical examination skills and procedures requires direct contact with patients (30–34).

Even though the short-term outcomes of the crisis management of educational activities at CMMS-AGU were encouraging, experts always recommend introspecting experience and based on which envisage a strategic plan to accomplish long term goals in terms of utilizing face-to-face and online methods (35, 36).

The aim of this study was to explore the overall experience of both medical students and faculty members of online and face-to-face learning, and their preference of the mode of learning (online, face-to-face, or blended) after the pandemic.

We expect that this will help us evaluate the experience of CMMS-AGU in the sudden shift from face-to-face to online learning during the COVID-19 pandemic, and to guide the teaching and learning practices in reforming the medical curricula to cater graduation of future-ready doctors.

## Materials and Methods

### Study Design

This is a mixed-method study with an exploratory two-phase design as we aimed to collect and analyze qualitative data to help explain and build upon the initial quantitative results. The study encompassed a quantitative component (researcher-made survey) and a qualitative component (focus group discussions for faculty members and medical students).

### Study Setting and Context

The study was conducted at the CMMS-AGU as part of a project to report the College's experience in dealing with the sudden digital transformation in higher education that was mandated by the COVID-19 pandemic. Before the pandemic, teaching/learning in lecture, tutorials, and labs as well as assessment practices were conducted completely face-to-face at the CMMS-AGU campus and its affiliated clinical settings. When the pandemic has struck, the College planned for a quick shift to the online mode and all teaching, learning, and assessment were conducted online during the pandemic.

### Participants

In the quantitative component, comprehensive sampling was used, where all full time faculty members (56 faculty members) who participated in teaching medical students both before and during the pandemic and all medical students (842 students) in years 2–6 of the medical program (171, 167, 165, 176, and 163, respectively), at the CMMS-AGU were invited to participate in this study through responding to the online survey. First year medical students who just joined the program were not included.

In the qualitative component, a purposive sample of 14 faculty members and 10 students participated in the focus group discussions (FGDs). Faculty who participated in the FGDs were nominated by the CMMS-AGU administration to represent all academic departments, while the selected students were originally the representatives of their batches in the students' council. Both were selected based on the assumption that they can provide in-depth and detailed information in relation the aim of the study.

### Tools for Data Collection

Data for the study was collected in the period from October to December 2020. Quantitative data in this study was collected through a unified survey form for both students and faculty members. The survey was drafted by the study team, based on review of relevant literature and other similar survey instruments (37–42). Every item was discussed in detail and changes were made wherever appropriate (addition, deletion, or editing of some statements). It was then transformed to an online form through Google Forms. The survey employed a 5-point Likert scale and consisted of 21 statements under three domains, which are: *Social Presence and Interaction, Collaborative Learning*, and *Satisfaction*. The respondents were asked to choose a response to each statement from strongly disagree to strongly agree, once for face-to-face learning and once for online learning. The participants were asked to rate their face-to-face learning based on their pre-pandemic experiences and rate their online learning based on their experience during the pandemic. They were told to consider this rating across all teaching activities (lectures, labs, tutorials …). In addition, one final question was added at the end of the survey where the students were asked to indicate their preferred mode of learning and the faculty members were asked to indicate which mode of learning might be more beneficial for the students after the pandemic: face-to-face, online, or blended. Blended learning was defined briefly in the question as “a mode of learning where students learn via both online as well as traditional, face-to-face ways.”

Validity of the survey was established through revision by three experts from the Medical Education Unit of the AGU. Based on the revision of the experts, modifications in some statements were made. Then the survey was revised one more time by the same experts and more modifications were made before the survey was made ready for distribution to the study participants. Examples of the modifications were adding a statement on the development of problem-solving skills (statement #12), changing statement #13 from “Collaborative learning in the courses is sufficient” to “Collaborative learning in the courses is effective”, adding a statement on learning environment (statement #20), and clarifying a few statements to prevent their equivocality (statements #5, 8, 9, 17, and 18).

For qualitative data collection, a total of four FGDs were conducted through Zoom™ (Zoom Video Communications, Inc., San Jose, CA, USA); two with faculty members only and another two with undergraduate medical students only. Mixing faculty members and students in the same FGD was avoided to prevent possible bias and nervousness, especially from the side of the students. Faculty members represented various department of the College of Medicine and Medical Sciences, and students represented various undergraduate batches. The participants were informed about the purpose of the study and a verbal consent was obtained. A semi-structured field guide was used to encourage discussion around topics identified through a literature search and opinion of the team. Development of the guide was informed by the quantitative findings. Several questions in the FGD guides were formulated based on the responses to the statements of the survey. Examples from students FGD guide were “How do you see face-to-face learning now? What do you miss in it and what don't you miss?” “Do you think online teaching/learning can replace face-to-face teaching/learning? Why? In case of yes, how do you think this can be?” “From your own experience, what should continue as online and why?” “From your own experience, what should continue as face-to-face and why?” and “Which method (online, face-to-face, or blended) of learning you generally prefer? Why?” Examples from faculty members FGD guide were “How do you see face-to-face learning now? What do you miss in it and what don't you miss?” “Have you found online learning to be helpful during the pandemic? In which way?” “From your experience, what benefits have you found in online learning? Give examples” “From your experience, what drawbacks/challenges have you found in online learning? Give examples,” and “Which method (online, face-to-face, or blended) of learning you generally prefer? Why?” The guide was reviewed and approved by the study team who comprised of three medical education experts. Each FGD extended over 45 min, and was conducted in English, recorded, and then transcribed.

### Data Analysis

Quantitative data analysis was done using the Statistical Package for the Social Science (SPSS) for Windows, version 27 (SPSS Inc., USA). Quantitative variables were presented as means and standard deviations. Paired samples *t*-test was used to compare between the mean response of faculty members and students on face-to-face and online learning. Chi-square test was used to compare the differences of responses of faculty members and students regarding their preference of online, face-to-face, and blended learning. A *p* < 0.05 was considered as a cut-off point of statistical significance.

For qualitative data analysis, three support staff members transcribed the recordings verbatim. We used thematic analysis method to code, interpret, and make sense of the data by using QSR NVivo version 12. The analysis was based on thematic analysis principles described by Braun and Clarke (43). Although a deductive approach was used to interpret data where we drew items from within the FGD guides, but an inductive approach was also used, where the themes – pattern within data answering our research objectives-were generated from within the data itself. One of the authors lead the analysis and first thoroughly read the text to familiarize with the data and then coded the text in NVivo. The codes were then checked again, revised, and merged into appropriate thematic ideas or categories by two more authors. In the next step, the data were interpreted, and themes were revised by refining their names to develop a construct that could best answer the research question. Themes were checked for internal homogeneity and external heterogeneity by reading the codes and a word search.

### Ethical Approval

Ethical approval was obtained from the Research and Ethics Committee of the College of Medicine and Medical Sciences, Arabian Gulf University.

## Results

The number of participants who responded to the survey was 33 faculty members out of the total of 56 (response rate was 59%) and 194 students out of 842 (response rate was 23%). Out of the 14 faculty members invited to participate in the FGDs, 13 attended and actively participated (response rate was 93%). Out of the 10 students invited to participate in the FGDs, 9 attended and actively participated (response rate was 90%).

Reliability of the survey was tested through Cronbach's alpha test and was found to be 0.98, which indicate excellent consistency of the survey.

The results are presented here in three sections as follows:

**Section I: Characteristics of Study Participants:**

Faculty members from all academic ranks (15.2% full professors, 30.3% associate professors, 30.3% assistant professors, and 24.2% lecturers) represented all the departments of the CMMS-AGU. Also, students represented all years of the medicine program (5.7% 2nd year, 20.6% third year, 24.2% 4th year, 11.3% 5th year, and 38.1% 6th year). First year students were not included as they were still freshmen who just came from high school and they did not experience the two modes of learning (online and face-to-face) at CMMS-AGU.

**Section II: Students' and Faculty Members' Responses to the Survey:**

Paired samples *t-*test was used to compare the differences of mean scores of responses of faculty members and students regarding both online and face-to-face learning. The results indicate that the mean scores of responses of both faculty members and students were higher for face-to-face learning that for online learning for all the survey statements. The differences were statistically significant (*p* < 0.05) for almost all the statements. The lowest scores were reported by the students in the areas related to interaction with other students and teachers, as well as the learning environment and its impact (statements 4, 6, 7, 8, 11, and 20) (Table 1). It is shown in the table that the averaged mean scores of faculty members in relation to the studied domains (Social Presence and Interaction, Collaborative Learning, and Satisfaction) and the Overall Experience are consistently higher than those of students for both online and face-to-face learning. The differences were statistically significant (*p* < 0.05).

**Table 1:** Comparison between responses of faculty and students on online and face-to-face learning.

**Statement**	**Faculty (*****n*** **=** **33)**			**Students (*****n*** **=** **194)**		
	**Online**	**Face-to-face**	***p-*value**	**Online**	**Face-to-face**	***p-*value**
	**Mean (±SD)**	**Mean (±SD)**		**Mean (±SD)**	**Mean (±SD)**	
1- Introductions between students and faculty at the beginning of the course create a sense of community	3.70 (±1.05)	4.73 (±0.52)	0.000*	3.24 (±1.41)	4.08 (±1.05)	0.000*
2- The instructors facilitate discussions in the sessions	4.18 (±0.88)	4.61 (±0.70)	0.024*	3.40 (±1.35)	4.14 (±0.95)	0.000*
3- Students' points of view are respected by their colleagues in the sessions	4.03 (±0.81)	4.45 (± 0.56)	0.008*	3.70 (±1.32)	4.24 (±0.87)	0.000*
4- Courses create a suitable environment for social interaction between students	3.30 (±1.07)	4.39 (± 0.75)	0.000*	2.88 (±1.49)	4.20 (±0.99)	0.000*
5- It is comfortable for students to interact in the sessions	3.73 (±1.04)	4.24 (±0.75)	0.036*	3.36 (±1.55)	3.89 (±1.17)	0.002*
6- The amount of interaction with other students in the sessions is appropriate	3.33 (±0.99)	4.30 (± 0.73)	0.000*	2.97 (±1.40)	4.01 (±1.04)	0.000*
7- The quality of interaction with other students in the sessions is appropriate	3.39 (±0.90)	4.30 (± 0.81)	0.000*	2.95 (±1.41)	4.01 (±1.06)	0.000*
8- Strong social relationships can be built during the courses	3.24 (±1.03)	4.30 (±0.98)	0.000*	2.46 (±1.35)	4.26 (±1.01)	0.000*
**Social presence and interaction**	**3.61 (±0.68)**	**4.42 (±0.60)**	0.000*	**3.12 (±1.16)**	**4.10 (±0.80)**	0.000*
9- The students can feel part of a learning community in the courses	3.97 (±0.68)	4.55 (±0.67)	0.000*	3.07 (±1.46)	4.32 (±0.91)	0.000*
10- The students can actively exchange ideas in the courses	3.94 (±0.97)	4.55 (±0.67)	0.003*	3.08 (±1.44)	4.18 (±0.94)	0.000*
11- The students can develop new skills and knowledge from other members in the courses	3.45 (±0.97)	4.30 (±0.85)	0.000*	2.97 (±1.47)	4.33 (±0.95)	0.000*
12- The students can develop problem-solving skills through peer collaboration during sessions	3.79 (±0.86)	4.45 (±0.71)	0.001*	3.11 (±1.45)	4.26 (±0.94)	0.000*
13- Collaborative learning in the courses is effective	3.76 (±0.66)	4.30 (±0.81)	0.002*	3.10 (±1.43)	4.19 (±1.00)	0.000*
14- Students save time with collaborative learning in the courses	3.85 (±0.80)	3.91 (±0.98)	0.763	3.54 (±1.49)	3.46 (±1.26)	0.658
15- Overall, collaborative learning experience in the courses is satisfying	3.64 (±0.74)	4.21 (±0.74)	0.001*	3.16 (±1.41)	3.93 (±1.12)	0.000*
**Collaborative learning**	**3.77 (±0.6)**	**4.32 (±0.67)**	**0.000**	**3.15 (±1.29)**	**4.10 (±0.86)**	0.000*
16- Students can learn effectively from the sessions	4.06 (±0.70)	4.33 (±0.65)	0.048*	3.29 (±1.51)	4.12 (±1.08)	0.000*
17- Students are stimulated to do additional reading or research on topics discussed in the courses	4.00 (± 0.79)	4.24 (±0.83)	0.058	3.37 (±1.46)	4.02 (±1.12)	0.000*
18- Discussions assist students in understanding other points of view	4.21 (±0.60)	4.42 (±0.66)	0.090	3.41 (±1.41)	4.19 (±0.95)	0.000*
19- The level of learning that takes place in the courses is of high quality	3.94 (±0.83)	4.30 (±0.81)	0.003*	3.17 (±1.52)	4.09 (±1.09)	0.000*
20- Learning environment in the sessions is motivating	3.76 (±0.83)	4.33 (±0.74)	0.001*	2.85 (±1.57)	3.99 (±1.17)	0.000*
21- Overall, the courses satisfy the students' learning expectations	3.70 (±0.81)	4.27 (±0.72)	0.000*	3.08 (±1.54)	4.08 (±1.05)	0.000*
**Satisfaction**	**3.90 (±0.78)**	**4.34 (±0.73)**	0.001*	**3.19 (±1.51)**	**4.08 (±1.08)**	0.000*
**Overall**	**3.76 (±0.89)**	**4.34 (±0.77)**	0.000*	**3.15 (±1.47)**	**4.09 (±1.05)**	0.000*

*Statistically significant.

Chi-square test of the preference of the mode of learning revealed a statistically significant difference between the preferences of faculty members and students (*p* < 0.05). More than half of the students preferred the face-to-face mode of learning, while most of the faculty preferred the blended mode of learning. On the other hand, only a small percentage of faculty members preferred online mode of learning compared to one third of the students (Table 2).

**Table 2:** Comparison of responses of faculty members and students regarding preference of the mode of learning.

**Mode of learning**	**Faculty members** **(*n* = 33)**	**Students** **(*n* = 194)**	**Chi^**2**^**	**Sig. (*p*-value)**
Online	1 (3.0%)	57 (29.4%)	47.8	0.000*
Face-to-face	12 (36.4%)	103 (53.1%)		
Blended	20 (60.6%)	34 (17.5%)		

*Statistically significant.

**Section III: Summary of Qualitative Results (FGDs):**

Qualitative analysis revealed five interrelated but distinct themes, namely: 1. Transforming the way theoretical teaching sessions are given, 2. Face-to-face teaching at campus cannot be replaced for some type of education, 3. Interaction in online sessions is limited, 4. Problems and challenges of online examinations, and 5. Technical issues and challenge of online education (Annex 1).

**Theme (1): Transforming the way theoretical teaching sessions are given:**

Faculty members in the FGDs proposed that at least 30% of the curriculum could be given online post-COVID-19 as it saves a lot of time and effort. Some aspects of the clinically oriented teaching including history taking and case discussions can also be delivered online in the future. For the subjects requiring teaching in a clinical setting or patient exposure, participants clearly indicated that student presence at the campus would be crucial to meet the learning objectives.

“*I can confidently say that 30–40% of the curriculum can be given online.”*

“*Subjects like Physiology and Biochemistry that can be taught online easily” (Faculty members – FGD 1)*.

Students believed that although online teaching was useful, and complemented their learning, they felt that it should be used as a standby plan for face-to-face teaching. The participants agreed that with the shift to online teaching during the COVID-19 pandemic, the students have most sessions recorded so they can watch them multiple times, which gives them time to absorb the ideas in the lecture.

“*I do not depend on the videos entirely. I first watch the lectures, and it is good because if you want to study something you can go back and watch it repeatedly until you understand it” (Student – FGD 2)*.

The participants also discussed the need to standardize and revamp the quality of the theoretical teaching sessions. The students reported that faculty members have a wide range of teaching approaches in the classroom and there was no one standard that everyone follows.

“*Some teachers explain very well and some of them (are just) ok, but I always have to go to watch a video for the complex concepts” (Student – FGD 2)*.

**Theme (2): Face-to-face teaching at campus cannot be replaced for some types of education:**

Most of the participants agreed that online teaching methods may not help achieve the intended learning outcomes compared to face-to-face teaching. A faculty member believed that responding to questions by students is easier during face-to-face teaching. Clinical faculty members confirmed that it was clear that clinical skills are difficult to be imparted online. They believed that the theoretical teaching is only a part of clinical training, while it would require students to be present and practically perform a procedure in front of their tutors to learn a particular clinical skill. Direct feedback on clinical examinations and procedures can only be given when they are conducted face-to-face in front of the tutors.

“*For surgical skills, like suturing, how can you teach this to students online? I can only give them the basic theoretical background online, but for the actual act and performance, you need to touch the patients or models physically” (Faculty member – FGD 1)*.

Similarly, faculty members and students unambiguously identified problems with the problem-based tutorial sessions conducted online, as they believed that these sessions are best conducted face-to-face with direct interaction between students with each other and students with their teachers. Apart from the claim that the problem-based tutorial sessions are useful face-to-face, the students believed that they are also more enjoyable because sitting physically with colleagues gives an opportunity to students to get to know and meet with their friends.

“*When it comes to the online tutorial sessions, I do not feel that it is that interactive or focused. I present and then I can go do something else away from the computer” (Student – FGD 2)*.

**Theme (3): Interaction in online sessions is limited:**

Students' perceptions of quality of theoretical teaching sessions they used to have face-to-face in the classrooms before the shift to the online mode during the pandemic showed that they value more interaction.

“*Using the pen and the smart screens and drawing on the white board in the classroom make the interaction very much better, valuable, and helpful and help us understand better” (Faculty member – FGD 1)*.

Faculty members and students discussed how valuable was interaction out of the classroom and that they missed talking to their peers since online teaching was implemented during the COVID-19 pandemic. Being present in the campus helps students interact with other students and build relationships that are difficult to be built online.

“*We used to meet and spend good time together, and personally, I used to study in the library with my friends. I see everyone studying and that encourages me to study as well” (Student – FGD 2)*.

Faculty members believed that nurturing communication skills in medical undergraduate students through physical interaction is crucial for them to be prepared to face patients in the future.

“*Medical students are not supposed to just acquire knowledge in online sessions, but they also need to learn communication skills through direct (face-to-face) interaction with their colleagues and teachers. They need that … they will face people and interact with patients” (Faculty member – FGD 1)*.

**Theme (4): Problems and challenges of online examinations:**

Online examinations were administered through an online student assessment application that supports remote proctoring and browsers lockdown. Although one version of each exam paper is prepared and used for all the students, presetting the application to shuffle the multiple-choice questions and their options makes the exam paper unique for each student. Faculty members were concerned about the quality of the examinations held online and whether the online examinations can properly assess students' knowledge. The first concern about examinations was whether the student knowledge has improved as reflected by the inflated marks they get in online examinations. Faculty members believed that the students were securing most of their marks in multiple-choice question-based examinations because these were much easier compared to on campus examinations conducted before. Another participant informed that almost one third of the students could secure full marks, which has never happened before. Participants justified the inflated results by the fact that the online examinations do not contain supply-type questions (where the students need to write the answers, not selecting them from a list of options as in selection-type questions) in which students used to lose marks. A faculty member believed that the inflated marks could also have resulted from the online assessment of some clinical skills, in which students received higher marks.

“*I do not believe that whatever the quality of the multiple-choice exams we prepare we can be confident that the real achievement of the learning outcomes by the students is guaranteed. Other question types and assessment methods are always needed” (Faculty member – FGD 1)*.

Students were concerned about the online examinations as they appeared to be dissatisfied with the time allocated for completing them, which is shorter than in the normal conditions where the exams are on campus and proctored. Another issue they identified was that they were not able to go back to a previous question to correct it whenever needed, a feature that is adjusted in online examinations application to prevent or reduce cheating possibilities.

“*In an exam, we were given 40 questions in 1 h, so we have to give each question one and a half minutes, which was not sufficient for answering some complex questions” (Student – FGD 1)*.

**Theme (5): Technical issues and challenges of online education:**

Communication technology issues were listed, by both the faculty members and students alike, as one of the main challenges of online education that took place in response to the sudden shift from face-to-face to online education. Initially, faculty members were recording their lectures through adding voice to the PowerPoint slides and sharing the recording with the students through cloud sharing platforms. Faculty members termed this initial interaction with students as “not actually virtual teaching” but rather a teaching based only on “recordings.” Challenges of recordings included issues with the length and quality of videos and voice as well as inability of the students to ask questions. Shortly, faculty members started to conduct synchronous live sessions with their students, where they could interact with them and could answer their questions and provide further instructions whenever needed. The important issue that popped up at that stage was the unstable internet speed that affected the live streaming of educational sessions. Faculty members and students reported that dealing with online education was not difficult, although the transition was not smooth.

“*I always make sure my students can hear me before I go on and on in the session. It is important to guarantee every single student is following instruction” (Faculty member – FGD 1)*.

## Discussion

This study employed a mixed method design and aimed at exploring the overall experience of both medical students and faculty members of online and face-to-face learning, and their preference of the mode of learning (online, face-to-face, or blended) after the pandemic. The study also explored the perceptions of participants regarding the improvements based on this experience that might enhance the learning experience in the post-COVID era.

Comparing the perception of the faculty members and students of face-to-face and online learning regarding the studied domains (Social Presence and Interaction, Collaborative Learning, and Satisfaction) and the Overall Experience revealed consistently higher mean scores for face-to-face learning than online learning. Outputs from FGDs support these results, where generally both faculty members and students preferred face-to-face learning over online learning. This is especially true when it comes to clinical and practical sessions, which is expected as physical examination skills cannot be learned without physical contact between students and real or simulated patients. The FGDs failed to suggest alternative strategies to replace face-to-face learning in this regard.

More than half of the students preferred face-to-face learning to online and even blended learning modes. This agrees with a pre-COVID study by Keis et al. (44), who reported higher satisfaction of their students by the face-to-face mode of learning. This also agrees with a recent study conducted during the pandemic and found that half of the students preferred face-to-face learning rather than online and blended learning (45). A recent study by Muthuprasad et al. (23) reported that most students showed a positive attitude toward online learning during the COVID-19 pandemic. One reason for that might be that online and blended learning modes are new to the students and they are not fully aware of the benefits of something they have not tried before. Another reason may be students' feeling that in a medical school everything should be taught face-to-face. However, a notable percentage of students in our study preferred online learning. This might be related to the fact that a big number of students in our college come from at least five countries to live and study in the college in Bahrain. Those students most probably prefer online learning because it allows them to study while they are at their home countries enjoying their families' significant moral and social support (46). Also, an explanation might be that students in online learning have access to more resources and they can study on their own pace.

In a pre-COVID study conducted on a large sample of Austrian students by Paechter and Maier (21), it was found that students appreciated online learning for its potential to provide a coherent structure of learning and supporting self-regulated learning. However, they preferred face-to-face learning for providing better communication and interaction, in addition to establishing better interpersonal relations and allowing for cooperative learning. This is clear from the lower mean scores of students' perception in most survey statements that are related to interaction, social relations, and learning environment.

On the other hand, most of the faculty members preferred blended learning. This is supported by the results of a study by Lapitan et al. (47), where they reported that a newly developed blended method of learning had positive impact on both teachers and students. This might be due to the flipped nature of blended learning, where the students can learn the theoretical part of lessons before coming to the classroom to do practical exercises facilitated by the teachers (48, 49). This is supported also by results from focus group discussions, where teachers reported that it would be better to mix between online learning for the theory parts of lessons (where students can do on their own pace before class) and face-to-face learning for deeper and more practical teaching in the classroom. However, more than one third of faculty members preferred face-to-face teaching. Reasons might be that this is the mode with which they are familiar and the fact that they were not ready for teaching online.

The five themes generated from the FGDs, in general, cover the different aspects of perceiving face-to-face and online learning by both faculty members and students and indicates a more positive attitudes toward face-to-face learning, which complements and confirms the results of the survey and analysis of students' performance in face-to-face and online exams, which indicates the consistency of the study results.

In theme 1 of the FGDs *(Transforming the way theoretical teaching sessions are given)*, faculty members believed that a notable part of the curriculum (theoretical part) can be taught online. This is supported by several recent studies (6, 50, 51) that reported adaptation and smooth transition to online teaching in theoretical content during the pandemic. Students also believed in the usefulness of online learning. This is congruent with the results of a number of studies that explored the perception of the medical students toward online learning during the pandemic (8, 52, 53). However, they think that online learning should better be kept as a standby option in case of crises. On the other hand, in Theme 2 of the FGDs *(Face-to-face teaching at campus cannot be replaced for some types of education)*, both faculty members and students reported that online cannot replace face-to-face teaching/learning when it comes to the clinical and practical skills components. In a similar study, Al-Balas et al. (54) found that distance learning represented a major challenge for acquiring adequate clinical skills. Also, Wallace et al. (55) found that remote learning of practical skills is inadequate and can be just a temporary alternative method of face-to-face teaching of these skills. However, in a pre-pandemic study, Gormley et al. (56) found that undergraduate medical students valued e-learning and rated it just as highly as other traditional methods of clinical skills teaching.

Regarding Theme 3 of the FGDs *(Interaction in online sessions is limited)*, both faculty members and students indicated the value of direct interaction between them inside and outside the classroom, which they missed in online learning. This is congruent with findings of a study by Wut and Xu (57), who reported poor student-to-teacher and student-to-student interaction in online settings. They added that interaction in traditional classrooms was important for the students to directly discuss with their classmates to obtain and exchange ideas, insights, and suggestions, which is quite difficult in online settings. The importance of that interaction is explained by the social presence theory (58).

Results in relation to Theme 4 of the FGDs *(Problems and challenges of online examinations)* indicated that faculty members perceived a few challenges in online examinations, most importantly the inflated marks gained by the students in online examinations (compared to usual traditional exam marks before the pandemic) which might not be indicative of the real achievement of the students. Such inflation may be explained by the higher possibility of cheating in online examinations, as was found by Jocoy and DiBiase (59), Michael and Williams (60), Lucky et al. (61), Harton et al. (62), and Chirumamilla et al. (63). However, it may also be explained by the decreased examination anxiety in online settings, as indicated by Stowell and Benett (64), or the use of innovative technologies and digital resources in distance learning, which made the students more confident and led to their better performance in online exams, as indicated by El Refae et al. (65). Concerns raised by the students in this regard were related to the shortened time of the online exams and the inability of the students to move freely between questions; strategies employed by the college to decrease the possibility of cheating.

Both faculty and students reported that technology issues were important challenges in online education, as indicated in findings from Theme 5 of the FGDs *(Technical issues and challenge of online education)*. Challenges faced by both faculty members and medical students in our study can be summarized in poor quality of recordings and unstable internet connection. This is supported by several studies that reported technology problems as important challenges that face using technology in education (23, 66, 67).

## Limitations

This study has some limitations. One of the limitations is the low response rate from the students. This might be because the survey was distributed online and there was not enough follow up with the students to complete the survey. However, the sample was fairly enough to give statistically valid results. Another limitation was that the study did not focus on the perception of the students of the imbalance between the theoretical and practical/clinical components during the pandemic due to the sudden shift to online learning which focused mainly on the theoretical content that can be easily taught online. A third limitation was that the study did not compare between the perception of students of different school years, which was due to insufficient samples from individual school years to give valid comparative results.

## Conclusions

The study revealed that although online learning is the possible educational adaptation during the pandemic, faculty and students still prefer face-to-face and blended learning. Qualitative analysis supported the quantitative results and revealed that both faculty and students agree on the benefits of online learning but prefer face-to-face and blended modes for their higher benefits. Educational adaptation in the form of online learning is obligatory during pandemics and suspension of traditional (face-to-face) education as an alternative to maximize the safety of all stakeholders and provide an easy and timely access to educational material and sessions, but this will not make such adaptation the future norm, especially in the study of medicine.

## Data Availability Statement

The raw data supporting the conclusions of this article will be made available by the authors, without undue reservation.

## Ethics Statement

The study was approved by Research and Ethics Committee of the College of Medicine and Medical Sciences, Arabian Gulf University (Ref. No.: E029-PI-12/20). Written informed consent for participation was not required for this study in accordance with the national legislation and the institutional requirements.

## Author Contributions

HA, MS, AK, AA-A, and AD: conceptualization, writing—review, and editing. HA, MS, AK, and JA: data curation. HA, AJ, and JA: data analysis. HA, MS, AA-A, AK, JA, and AD: methods. HA, MS, AK, AJ, JA, AA-A, and AD: writing—original draft. All authors have reviewed and approved the manuscript.

## Conflict of Interest

The authors declare that the research was conducted in the absence of any commercial or financial relationships that could be construed as a potential conflict of interest.

## Publisher's Note

All claims expressed in this article are solely those of the authors and do not necessarily represent those of their affiliated organizations, or those of the publisher, the editors and the reviewers. Any product that may be evaluated in this article, or claim that may be made by its manufacturer, is not guaranteed or endorsed by the publisher.

## Figures and Tables

**Annex 1:** List of codes and categories forming the final themes in the qualitative analysis.

**Codes**	**Categories**	**Themes**
• Improving the quality of theoretical lessons. • Participation in online resources has improved. • Recorded theoretical teaching sessions are preferred. • Teaching that should be given online. • Attending from home is preferred. • Future is for blended learning. • Time and effort saved in online learning. • Online learning complements face-to-face. • Dressing up and surroundings for online sessions.	• Teaching that should be given online. • Time and effort saved in online learning. • Recorded theoretical teaching sessions are preferred. • Improving resource session quality.	***Theme 1:*** *Transforming the way theoretical teaching sessions are given*.
• Face-to-face teaching/learning has its limitations. • Communication is better built with face-to-face learning. • Professional (practical) skills training must be given at the campus. • Direct participation in face-to-face is better than in online.	• In-person participation in face-to-face is better than in online. • Communication is better built with face-to-face learning. • Clinical training, laboratory and professional (practical) skills, and tutorials must be conducted face-to-face.	***Theme 2:*** *Face-to-face teaching at campus cannot be replaced for some types of education*.
• Quality of presentations in face-to-face teaching. • Interaction in sessions. • Student's presence in the sessions. • Attending campus is much better.	• Interaction in sessions. • Students' presence in online sessions. • Valuing the direct human interactions and socializing.	***Theme 3:*** *Interaction in online sessions is limited*.
• Achievement in assessments. • Achieving the transparency in online examinations. • Setting online examinations is challenging.	• Challenges of online examination. • Assessment issues.	***Theme 4:*** *Problems and challenges of online examinations*.
• Initial challenges to going online. • Technical issues and support required.	• Initial problems to go online. • Support required for technical issues.	***Theme 5:*** *Technical issues and challenges of online education*.
